# Supplementary material for: Association of Insulin-like Growth Factor-1 with Bone Mineral Density in Survivors of Childhood Acute Leukemia
Source: Cancers (Basel). 2024 Mar 27;16(7):1296. doi: 10.3390/cancers16071296 (PMC11011034; doi:10.3390/cancers16071296)
Supplement: Supplementary file 1 [file cancers-16-01296-s001.zip › cancers-2928890-supplementary.pdf]

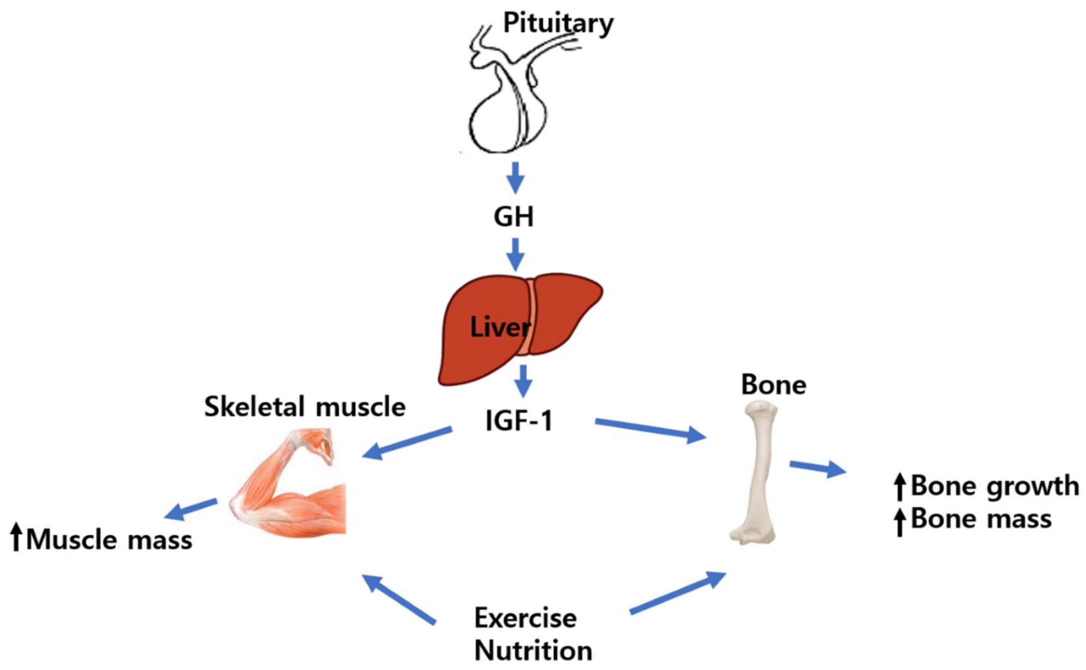

Supplementary Figure S1. Role of GH/IGF-1 axis and lifestyle on bone health. GH, growth hormone; IGF-1, insulin-like growth factor.
